# Profiles of miRNA Isoforms and tRNA Fragments in Prostate Cancer

**DOI:** 10.1038/s41598-018-22488-2

**Published:** 2018-03-28

**Authors:** Rogan G. Magee, Aristeidis G. Telonis, Phillipe Loher, Eric Londin, Isidore Rigoutsos

**Affiliations:** Computational Medicine Center, Thomas Jefferson University, 1020 Locust Street, Philadelphia, PA 19107 USA

## Abstract

MicroRNA (miRNA) isoforms (“isomiRs”) and tRNA-derived fragments (“tRFs”) are powerful regulatory non-coding RNAs (ncRNAs). In human tissues, both types of molecules are abundant, with expression patterns that depend on a person’s race, sex and population origin. Here, we present our analyses of the Prostate Cancer (PRAD) datasets of The Cancer Genome Atlas (TCGA) from the standpoint of isomiRs and tRFs. This study represents the first simultaneous examination of isomiRs and tRFs in a large cohort of PRAD patients. We find that isomiRs and tRFs have extensive correlations with messenger RNAs (mRNAs). These correlations are disrupted in PRAD, which suggests disruptions of the regulatory network in the disease state. Notably, we find that the profiles of isomiRs and tRFs differ in patients belonging to different races. We hope that the presented findings can lay the groundwork for future research efforts aimed at elucidating the functional roles of the numerous and distinct members of these two categories of ncRNAs that are present in PRAD.

## Introduction

The rapidly decreasing cost of next generation sequencing has enabled the unbiased profiling of short and long non-coding RNAs (ncRNAs) in many settings. This has facilitated the quantification of select RNAs under a variety of conditions, the discovery of new categories of transcripts, and the identification of new members of previously established categories^1,2^. In this report, we focus on two specific categories of ncRNAs: the isoforms of microRNAs (miRNAs), or *isomiRs*, and fragments that are derived from transfer RNAs (tRNAs), or *tRFs*. We examine the profiles of isomiRs and tRFs in the PRAD samples that are available in the TCGA repository.

MiRNAs are ~22 nucleotide (nt) long non-coding RNAs^3^ that derive from hairpin-shaped precursor transcripts. They promote the degradation of sequence-matched mRNA targets or inhibit their translation^4–8^. The precursors of miRNAs were initially thought to produce at most a single mature product from each hairpin arm^9,10^. It is now known that miRNA precursor arms give rise to many distinct isoforms, termed “isomiRs”^11,12^. Any two isomiRs from the same miRNA arm typically differ in either their 5′ termini, 3′ termini, or both. In previous work, we showed that the most abundant isoform is frequently different from the one annotated as the “canonical” or “archetypal” sequence^13^ that is found in public databases like miRBase^9,14^. 

In previous work, we analyzed hundreds of transcriptomic datasets from LCL samples and showed that isomiRs are produced constitutively and are differentially abundant among people in a manner that depends on the individuals’ sex, population origin, and race^13^. We also showed that similar isomiR dependencies exist in the context of breast cancer and demonstrated experimentally that isomiRs from the same hairpin arm can target largely distinct sets of mRNAs^15^. We also examined whether isomiR profiles differ across cancer types. We analyzed 10,271 cancer samples from TCGA that represent 32 cancer types and found that by simply examining whether an isomiR is present or absent in a sample, we could build highly sensitive and specific biomarkers that distinguish among the 32 cancer types^16^. In the case of PRAD, we showed that isomiRs can correctly classify TCGA datasets (obtained by the Illumina platform) as well as datasets obtained from two other platforms (Affymetrix, and ABI SOLiD)^16^.

tRFs represent a new class of short regulatory non-coding RNAs that were recently discovered through analysis of short RNA sequencing data^17–19^. tRFs are relatively short RNA oligonucleotides, with lengths ranging from 18 to ~34 nt. In this presentation, we study only tRFs that overlap with mature tRNAs. These can be grouped into five classes^17,18,20–22^: 5′-tRFs, i-tRFs, 3′-tRFs, 5′-tRNA halves (5′-tRHs), and 3′-tRNA halves (3′-tRHs).

Several proteins have been implicated in the production of tRFs. Chronologically first, Angiogenin was shown to be a producer of tRNA halves under stress conditions^17,21–23^. RNAse Z produces tRFs derived from premature tRNA transcripts^24^. Finally, the miRNA-producing enzyme Dicer has also been implicated in the production of tRFs in several species^24–27^, but many tRFs are also produced independently of Dicer^27^. In complete analogy to isomiRs, we showed that tRFs are produced constitutively and are differentially abundant among people in a manner that depends on the individuals’ sex, population origin, and race as well as on tissue, and disease subtype^20^. Functionally, tRFs have also been reported to increase in abundance after hepatitis B and C infection^28^, to act as miRNAs^26^, to act as molecular decoys of an RNA binding protein^29^, to mediate neuroprotective responses^30^, to inhibit angiogenesis after ischemic injury^31^, and to be involved in sex-hormone pro-proliferative signaling^23^. Collectively, these results suggest important functional roles for these molecules.

Recent work by the TCGA PRAD Consortium has improved our understanding of the biochemistry of prostate cancer subtypes^32^, but many questions regarding the biochemical progression of the disease remain unanswered^33–37^. Several of the currently open questions include the identification of new and potentially actionable biochemical targets for treatment, the relative merit of treatment vs. active surveillance^38^, the relevance of race in establishing risk, and the impact of race in disease prognosis^33,39,40^. To date, and to the best of our knowledge, the significance of isomiRs and tRFs has not been studied systematically in PRAD.

In this study, we characterize the expression of isomiRs and tRFs in the context of PRAD. Specifically, we investigate the possibility of associations between clinical categories of PRAD and the expression of isomiRs and tRFs, and report on whether these ncRNAs are associated with disease progression.

## Results

### Patients, samples, and sequencing limitations

526 samples were obtained from 472 patient donors. The patients span a range of ages and several races (Table 1). These samples form evenly sized subgroups from the standpoint of Gleason score at initial diagnosis (Table 1). It is important to note here that short RNA-seq for the TCGA project was capped at 30 cycles of sequencing. This choice does not pose a problem for miRNAs and their isoforms, because their typical length is ~22 nt. However, in the case of tRFs, the 30-cycle limit prevents us from identifying and quantitating longer molecules, such as the 5′-tRHs and 3′-tRHs^41^.

**Table 1:** Clinical characteristics of prostate cancer samples. 526 white-listed samples were analyzed from The Cancer Genome Atlas collection of patients with prostate cancer. 472 patients contributed these samples. Race characteristics as reported in TCGA clinical patient data Biotab files downloaded on January 29, 2016 are shown. Also shown are age characteristics and the Gleason grade by histological examination.

**Sample Characteristics (n = 526)**				
Race	White	431	Normal Tissue < 6	50
	Black_or_African_American	63	Tumor Tissue	476
	Asian	12	Gleason Score	
	American_Indian_or_Alaska_Native	1	3 + 3, 2 + 4	48, 1
	[Not_Available]	19	3 + 4	143
Age at diagnosis	40–49	27	4 + 3	93
	50–59	166	4 + 4, 5 + 3, 3 + 5	54, 6, 8
	60–69	230	4 + 5, 5 + 4	67, 45
	70+	49	5+5	11

### Prostate isomiRs - summary statistics

**Figure 1:**
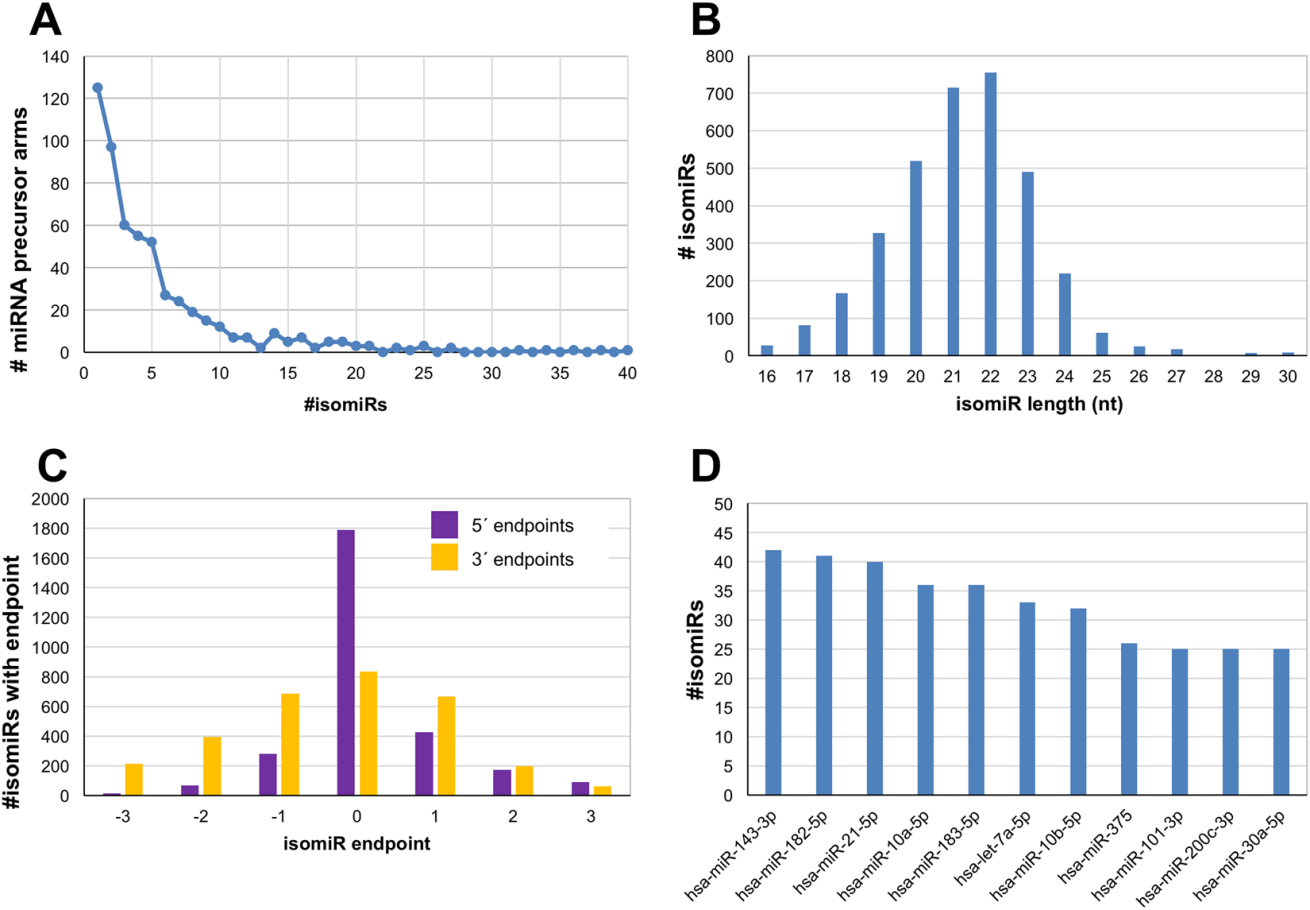
Classification of isomiRs by mature miRNA, length and endpoint. We collected 3,178 isomiRs from the pool of 526 TCGA PRAD samples. (**A**) The number of mature miRNAs which give rise to N isomiRs (N ranges from 1 to 40). (**B**) The number of isomiRs of each specific possible length from 16–30 nt within these data. (**C)** The number of isomiRs having different 5′ and 3′ endpoints with respect to the archetype miRNA’s endpoints. Note that this panel is restricted and shows only endpoints that differ by at most 3 nt from the endpoints of the archetype. (**D**) The top-11 miRNA loci producing 25 or more isomiRs.

For each sample in turn, we used Threshold-seq^42^ (see Methods) to automatically determine a dataset-dependent, adaptive threshold. Using these thresholds across the 526 datasets, we identified 3,178 isomiRs that were used in the subsequent analyses. Each of these 3,178 isomiRs exceeded Threshold-seq’s threshold in at least one of the analyzed datasets. 3,104 of these isomiRs are unambiguously annotated in our analyses, i.e. they map to a single mature miRNA or miRNA precursor, whereas the remaining 74 map to a total of 28 miRNA loci with known paralogues in the human genome. The 3,104 isomiRs arise from 600 distinct miRNA precursor arms (Fig. 1A) that correspond to 391 unique miRNA loci.

The isomiRs in TCGA PRAD have lengths that are typical of mature miRNAs. The most frequently encountered isomiR length is 22 nt, followed closely by 21 nt (Fig. 1B). Moreover, the endpoints of isomiRs (Fig. 1C) follow essentially the same distributions that we reported in our prior work^15^. We note that nearly one half (43.0%) of all identified isomiRs have 5′ termini that differ from the 5′ termini of the miRNA sequences found in miRBase, in agreement with what we found previously in a different tissue^13^. IsomiRs with different 5′ termini have different seed sequences compared to the archetype in miRBase, and, thus, different mRNA targets than the archetype^15,43–45^. Consequently, the identification of so many isomiRs with 5′ termini is of high potential importance, as it suggests the existence of a large repertoire of genes targeted by miRNAs that have not been explored to date. Moreover, 73.3% of the isomiRs have 3′ termini that differ from the 3′ termini of the corresponding archetypes found in miRBase.

Across all miRNA precursors, a few arms appear to be the greatest producers of distinct isomiRs in the PRAD context: 42 isomiRs are produced from miR-143-3p, 41 from miR-182-5p, 40 from miR-21-5p, 36 from miR-183–5p, 36 from miR-10a-5p, and 33 from let-7a-5p. (Fig. 1D). Supp. Table 1 lists all of the mature miRNAs that produce more than one isomiR above threshold in at least one of the analyzed datasets.

### Prostate tRFs – summary statistics

We mined the sequenced reads using our MINTmap tRF mining method^41^, then used the Threshold-seq method^42^ to determine which tRFs to retain for each dataset. Across the 526 TCGA PRAD datasets, we identified 6,296 distinct tRFs that pass threshold in at least one dataset. Of these, 3,903 (62.0%) arise from nuclearly-encoded tRNAs, whereas the remaining 2,393 (38.0%) arise from mitochondrially-encoded tRNAs. In other words, the 22 mitochondrial (MT) tRNAs contribute almost half as many unique tRFs as do the 610 nuclear tRNAs, a rather unexpected finding. 140 tRFs show non-zero expression across all 526 (100%) datasets, which suggests possible relevance of tRFs for PRAD biology. Moreover, 83 tRFs show a normalized mean abundance at least as high as 10 reads per million (RPM), in either normal or prostate cancer samples.

### Prostate tRFs – lengths, structural categories, source isoacceptors

**Figure 2:**
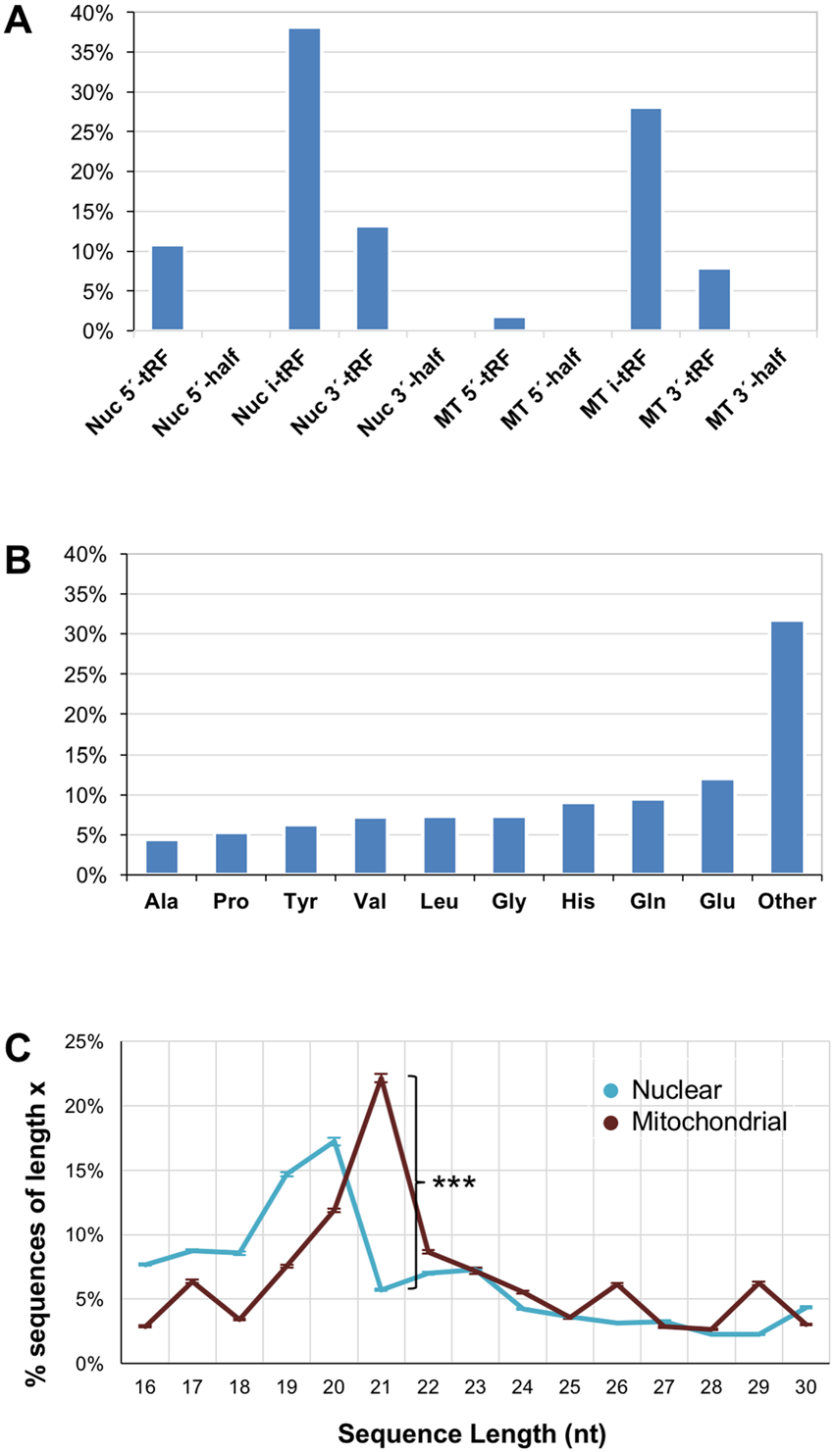
Classification of tRFs by isodecoder and genome of origin. (**A**) Percentage of tRFs that map to a specific structural category (nuclearly- and MT-derived tRFs are shown separately). (**B**) Percentage of tRFs that arise from isodecoders of specific amino acids. (**C**) Distribution of tRFs as a function of length (nuclearly- and MT-derived tRFs are shown separately). The whiskers represent standard error of the mean  across samples. Nuc: nuclear. MT: mitochondrial.

The majority (66%) of the 6,296 tRFs are i-tRFs^20^, i.e. they overlap the interior of the mature tRNA sequence. Interestingly, nearly one half (42%) of all i-tRFs are produced by MT tRNAs, a disproportionately high contribution considering that there are only 22 MT tRNA sequences. The remainder of the tRFs are almost evenly split among nuclear 5′-tRFs, nuclear 3′-tRFs, and mitochondrial 3′-tRFs (Fig. 2A). Interestingly, five isoacceptors contribute 44% all of the tRFs we identified in PRAD. They are: Leucine (Leu), Glycine (Gly), Histidine (His), Glutamine (Gln), and Glutamic acid (Glu) (Fig. 2B). The lengths of nuclearly-encoded tRFs differ characteristically from those of MT-encoded tRFs (Fig. 2C). This is similar to what we observed previously in other human tissues^20^. Nuclearly-encoded tRFs appear to favor shorter lengths (16–20 nt), whereas mitochondrially-encoded tRFs tend to favor specific and generally-longer lengths (17, 21, 26, and 29 nt) (Fig. 2C).

### Prostate isomiRs, prostate tRFs, and comparisons to clinical characteristics

#### Tissue state

**Figure 3:**
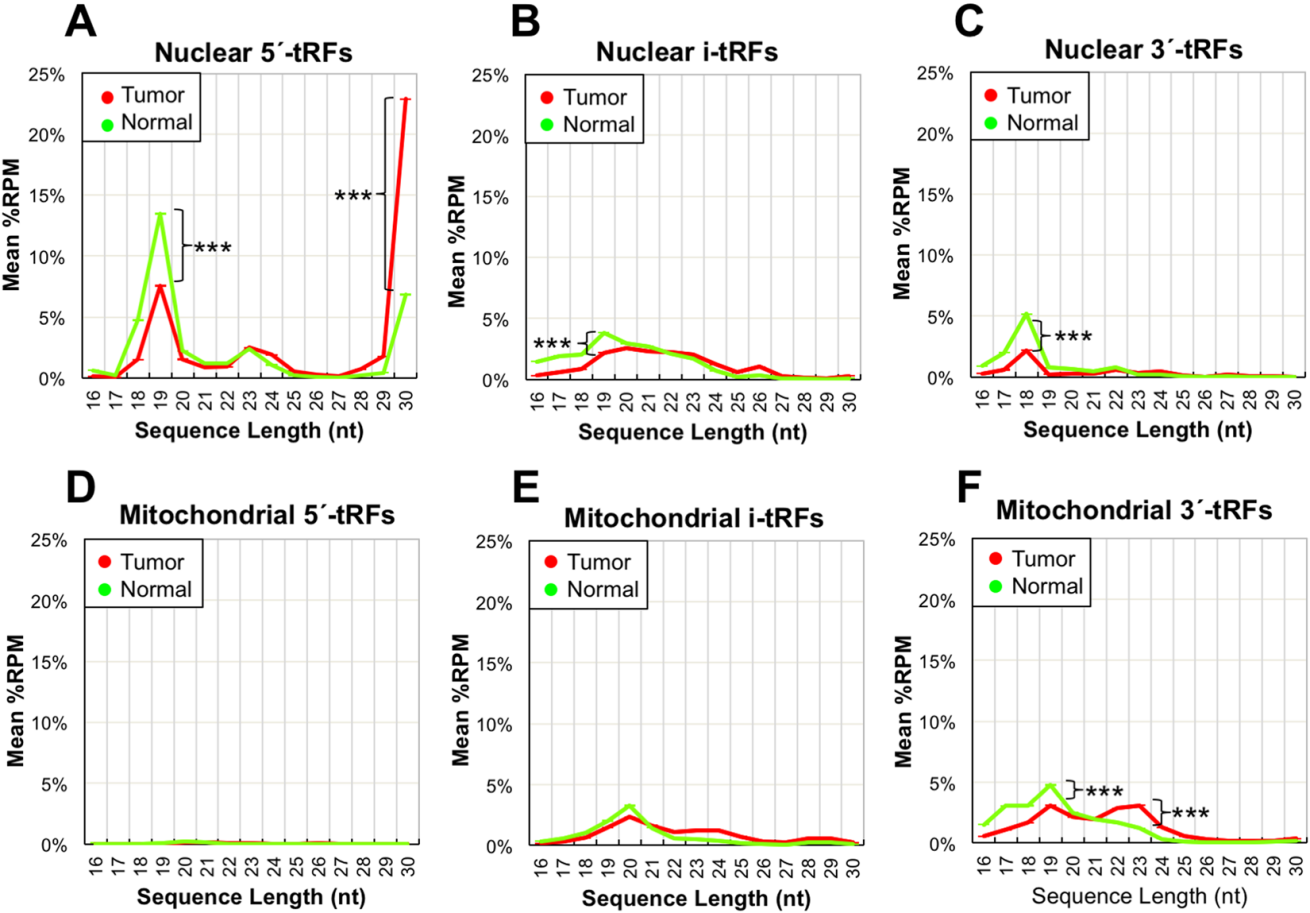
Classification of tRFs by length and structural category, in normal and tumor. (**A–C**) Nuclear tRFs. (**A**) Distribution of 5′-tRFs arising from nuclear tRNAs. 5′-tRFs begin at either the −1 or the +1 position of the mature tRNA. (**B**) Distribution of i-tRFs from nuclear tRNAs. i-tRFs begin after the +1 position and terminate within the mature tRNA sequence. (**C**) 3′-tRFs from nuclear tRNAs. These tRFs begin within the mature tRNA and end within the post-transcriptionally-added CCA. (**D–F**) The counterpart distributions for 5′-tRFs, i-tRFs, and 3′-tRFs arising from mitochondrial tRNAs. In all plots, the distributions of tRFs in normal (tumor, respectively) samples are shown in green (red, respectively).

When we compared tumor and normal samples, we observed marked differences in the lengths of tRFs from the structural categories described above. We computed density functions for the probability of encountering a tRF of length X in each structural category, and included genome of origin (nuclear or mitochondrial) as an additional variable. For several lengths, we found statistically-significant (Mann-Whitney U-test) differences between normal and tumor. Figure 3 shows these findings. In it, a few representative cases are highlighted in the plots with asterisks. Individual differences between structural categories appear to be specific to the tissue and tissue-state combination. For example, in TCGA’s breast cancer (BRCA) samples, we found short (18–20 nt) 5′-tRFs to be more prevalent in the *tumor* samples^20^. Unlike BRCA, in TCGA PRAD, 5′-tRFs with these lengths are more prevalent in the *normal* samples. Additionally, 3′-tRFs also tend to be short (18–20 nt) in normal prostate samples, which is again unlike what we found in normal breast. Finally, we note that 3′-tRFs from mitochondrially-derived tRNAs differ between normal prostate and tumor by length: short (19 nt) tRFs are more frequent in normal samples, whereas longer (23 nt) tRFs are more frequent in prostate tumor samples.

#### Tissue state and patient race

**Figure 4:**
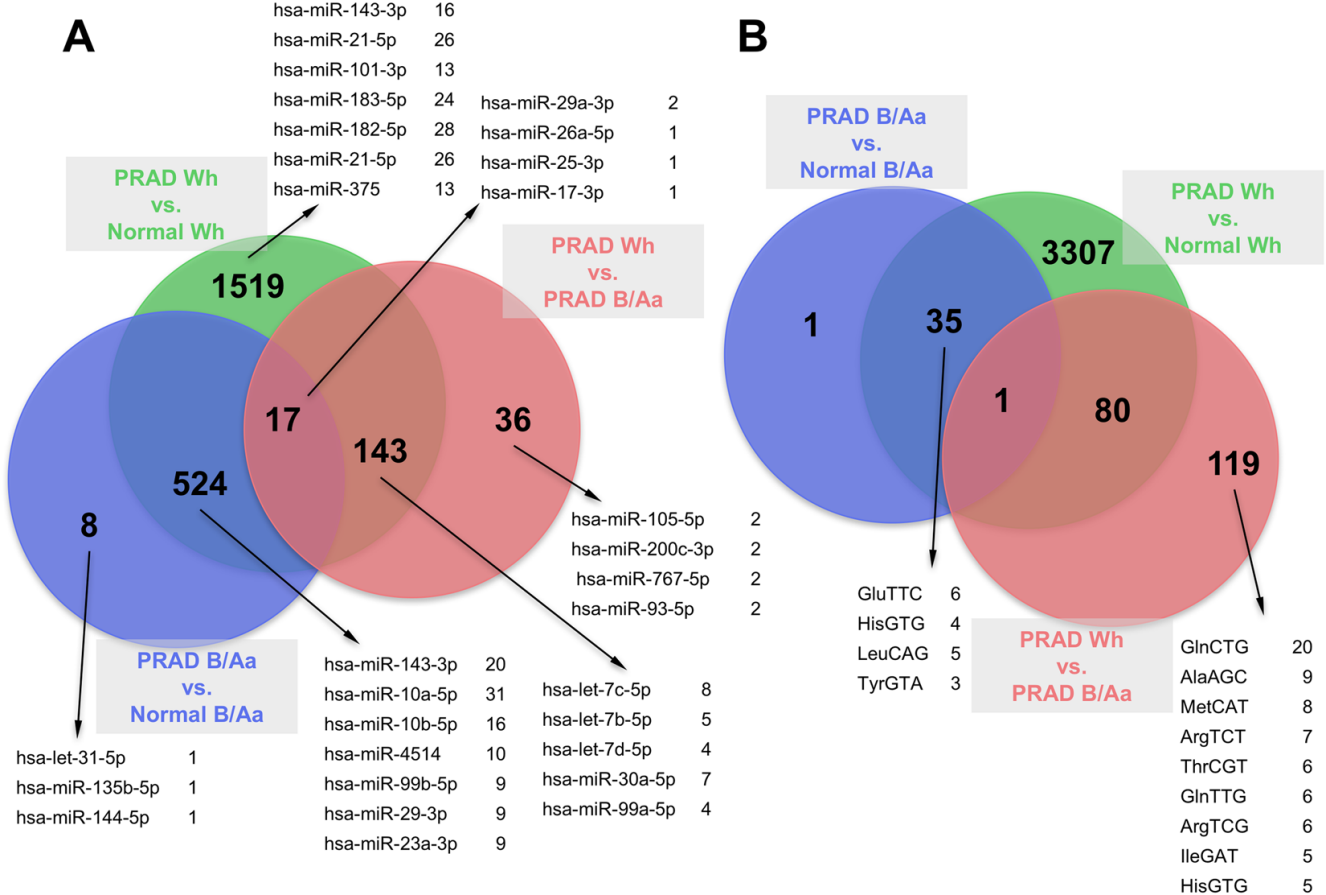
Differentially abundant isomiRs and tRFs in prostate cancer. We used SAM to determine which tRFs and isomiRs are differentially abundant at an FDR ≤ 5% in four comparisons: PRAD Wh vs. Normal Wh, PRAD B/Aa vs. Normal B/Aa, and PRAD Wh vs. PRAD B/Aa. (**A**) isomiRs. (**B**) tRFs. Representative molecules from the corresponding group of regulators are also listed. We found no isomiRs or tRFs that are differentially abundant between Normal Wh and Normal B/Aa, so these groups are not plotted.

We next used Significance Analysis of Microarrays (SAM)^46^ to compare the abundance of isomiRs and tRFs, respectively, across tissue states (both races) and across tissue states within each race group in turn. We retained only isomiRs or tRFs that were differentially abundant and had an associated False Discovery Rate (FDR) of 5% (Supp. Table 2). Of 3,178 isomiRs, 524 are differentially abundant between PRAD and normal prostate in *both* Black or African American patients (B/Aa) and White (Wh) patients (Fig. 4A). A larger number of isomiRs (1,519) are differentially abundant in PRAD vs. normal comparisons, exclusively in Wh patients. We also analyzed the 6,296 tRFs and found 35 tRFs to be differentially abundant between PRAD and normal in *both* Wh and B/Aa patients (Fig. 4B). A much larger portion comprising 3,307 tRFs is differentially abundant exclusively in Wh patients. Interestingly, 119 tRFs are exclusively differentially abundant between PRAD Wh and PRAD B/Aa.

#### Gleason score and prognostic group

In order to determine whether the profiles of tRFs or isomiRs are associated with progression of disease, we performed multi-class SAM analysis with the Gleason score and the newly-proposed five-grade prognostic system (Supp. Table 2)^47^. We used only samples for which Gleason score agreed in both clinical and pathologic grading (n = 518 samples), and also kept normal samples (with Gleason score lower than 6) in the comparisons. In these analyses, we maintained the FDR threshold at 5%. We found that n = 2,441 isomiRs and n = 4,331 tRFs are differentially abundant between Gleason-score subgroups. We find that n = 2,493 and 4,652 isomiRs and tRFs are differentially abundant when we instead reclassify tumors using the alternative five-grade system. Importantly, the identified groups of isomiRs and tRFs are highly congruent: the same n = 2,401 isomiRs and n = 4,187 tRFs arise from both of these two independent analyses. Though prognostic groups are based on Gleason score, the subgroups do differ in that the samples of Gleason score 7 are split into two categories, each of which is characterized by a different prognosis. The respective isomiR and tRF profiles do differ between these two groups. All of these isomiRs and tRFs are shown in Supp. Table 2.

### Using the abundance of all isomiRs from a miRNA locus as a proxy for isomiR profiles

Quantitative RT-PCR (qRT-PCR) methods such as Taqman miRNA qPCR and Exiqon LNA miRCuRY are designed with an eye towards distinguishing among miRNAs that have base differences that are internal to the target sequence. The ability of these methods to distinguish isoforms that differ at their endpoints has been a topic of scientific debate. In recent work, we examined both Taqman miRNA qPCR and Exiqon LNA miRCuRY and found that these methods cannot distinguish end-point isoforms, in either synthetic or true cellular contexts^48^. While we experimented with isomiRs only, we expect that these findings extend to tRFs as well. Our findings suggest that SYBR Green-based methods are also affected. This includes variants of the SYBR Green method such as the one used in a previous B-cell lymphoma study^26^, which is similar in spirit to the LNA miRCuRY method, but without the latter’s additional constraints. Importantly, neither the SYBR Green variant nor the LNA miRCuRY can guarantee the specific 5′ and 3′ endpoints of the target.

**Figure 5:**
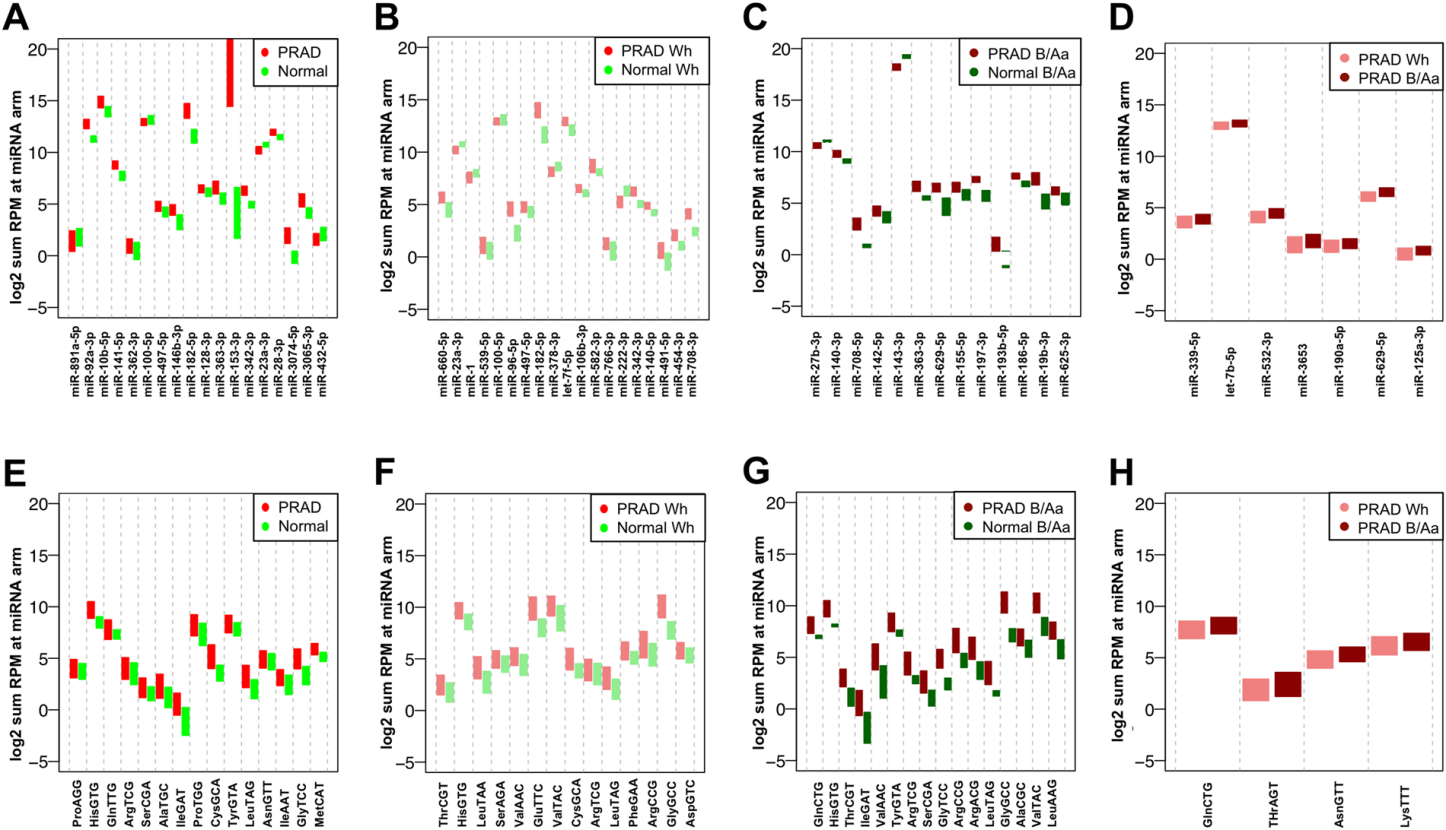
Comparison of total RPM at miRNA arms and mature tRNA loci. Separately for each sample, we summed the RPM values of each isomiR that arises from a specific locus and assigned the resulting value to that locus. There were 628 loci that produced isomiRs in various combinations in the 526 samples. At most 20 of the miRNA-arms exhibiting differential abundances are shown in each case, if available. (**A**) PRAD vs. normal (all patients). (**B**) PRAD vs. normal (Wh patients only). (**C**) PRAD vs. normal (B/Aa patients only). (**D**) PRAD from Wh patients vs. PRAD from B/Aa patients. (**E–H**) counterpart plots to (**A–D**) for isoacceptors. Analogously, we summed the RPM values of each tRF that arises from a given isoacceptor, and assigned the resulting value to the isoacceptor. There were 47 isoacceptors that produce tRFs in various combinations in the 526 samples. At most 20 of the isoacceptors exhibiting differential abundances are shown in each case, if available.

In light of this limitation, we carried out a second analysis of the deep sequencing data where we aggregated the abundance of all isoforms from a given miRNA arm into a single measure. Starting with the pool of isomiRs, and for each sample in turn, we summed the RPM values of all isomiRs from each annotated miRNA arm that was transcribed. This resulted in 628 observations per sample. Figure 5A–D show boxplots for several miRNA loci that showed significant differences between clinical groups (Welch’s independent t-test, p-value ≤ 0.05). In each plot, the statistical significance of the shown miRNA arm decreases from left to right. Not more than the top 20 most significant comparisons are shown in each case.

### Using the abundance of all tRFs from a tRNA isoacceptor as a proxy for tRF profiles

As we mentioned in the previous section, the current commercially available quantitative methods that target specific molecules cannot accurately determine the abundance of isomiRs or tRFs. Thus, we repeated the above re-analysis, this time for tRFs, wherein we summed up the RPM values of tRFs from the same isoacceptor. This resulted in 47 observations per sample. Figure 5E–H show the resulting boxplots (Welch’s independent t-test, p-value ≤ 0.05). Again, the statistical significance of the shown isoacceptors decreases from left to right. Not more than the top 20 most significant comparisons are shown in each case.

### Correlation and anti-correlation of the abundance of prostate isomiRs

**Figure 6:**
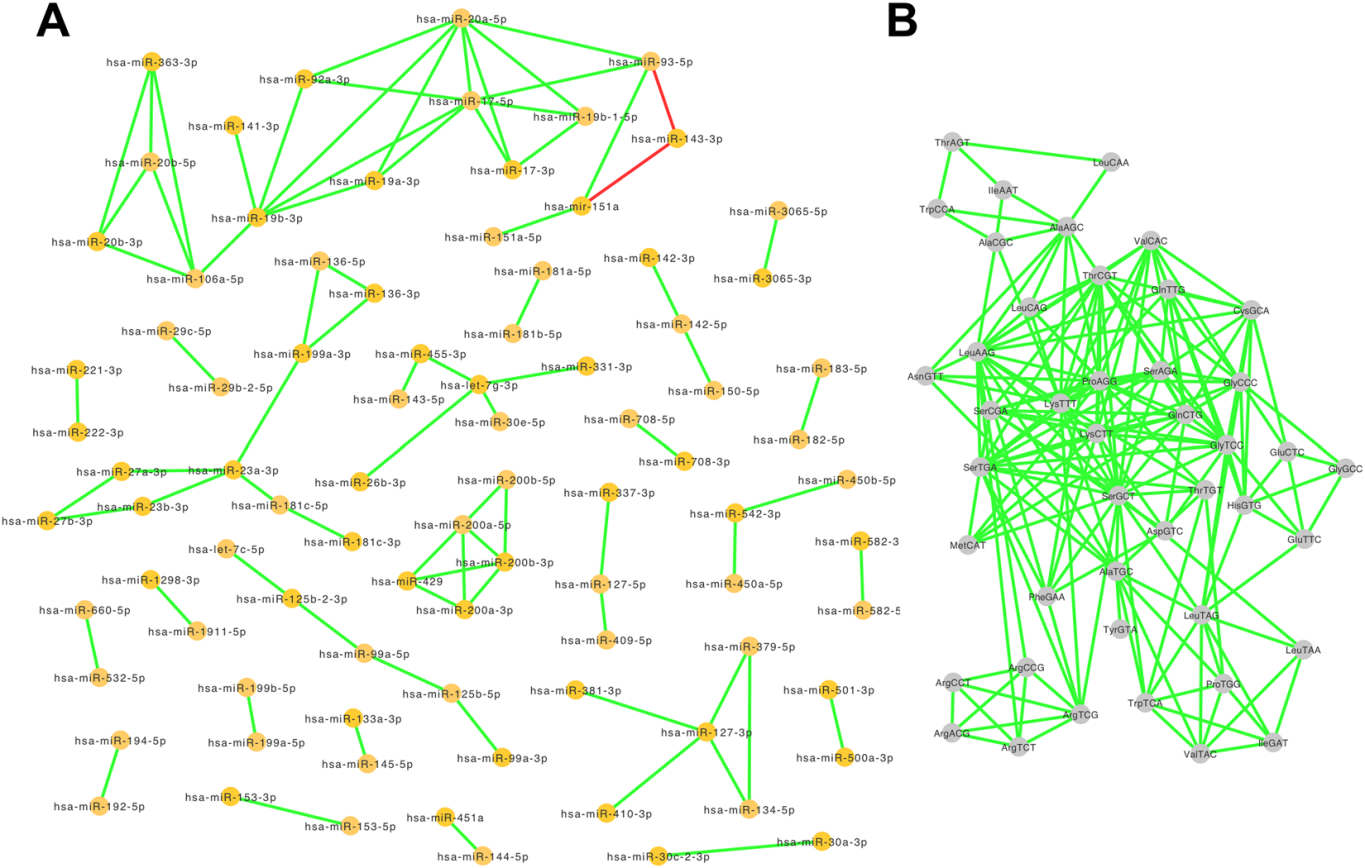
Networks of isomiR-isomiR and tRF-tRF correlations. We collapsed isomiR (tRF, respectively) abundance to the corresponding miRNA-arm (tRNA isoacceptor, respectively), and computed Spearman correlations among the resulting observations. (**A**) isomiR-isomiR correlations represented by the corresponding miRNA arms. Gold nodes represent miRNAs. (**B**) tRF-tRF networks as captured by the corresponding isoacceptors. In both panels, only correlations with an absolute value ≥ 0.75 and an FDR ≤ 5% were retained and shown. Green edges: positive correlations. Red edges: negative correlations.

We also determined how well the levels of individual miRNAs correlate with each other in the context of PRAD tumors. First, we examined correlations at the level of the mature miRNA locus. In this case, the abundances of isomiRs from a miRNA locus were summed into a single number (see above). We computed the Spearman correlation for all 628 miRNA arms that produced one or more isomiRs across the 526 PRAD datasets. We only retained correlations if their absolute value was ≥0.75 and FDR ≤ 5% (Supp. Table 3). Figure 6A shows the correlations/anti-correlations at the level of the miRNA locus, which survive this filtering. Each node in the network represents an individual miRNA arm that produces at least one isomiR. Green edges indicate positive correlations, whereas red edges indicate negative correlations. Supp. Table 3 also lists all isomiR-isomiR correlations whose absolute value was ≥0.75 and FDR ≤ 5%.

### Correlation and anti-correlation of the abundance of prostate tRFs

We repeated the correlation analysis using tRFs this time. As with the miRNAs/isomiRs, we carried out the computations in two ways. First, we computed correlations using the summed abundances for tRFs from the various tRNA isoacceptors that are active in PRAD (see above). Second, we computed correlations using the individual tRFs. As before, we retained only Spearman correlations with absolute value ≥ 0.75 and FDR ≤ 5%. Figure 6B shows the correlations/anti-correlations at the level of the tRNA isoacceptor, which survive this filtering (see Supp. Table 3 for the complete list). Each node represents an isoacceptor that produces at least one tRF. Green edges indicate positive correlations. Supp. Table 3 also lists all tRF-tRF correlations that survive the correlation and FDR filtering.

### Correlation between isomiRs and mRNAs

To investigate the possibility of regulatory networks involving isomiRs and mRNAs, we selected mRNAs with a mean value of reads per thousand mapped reads (RPKM) value that was at most 10 log_2_ units from the most abundant observed value for ACTB across the 526 samples. By design, these mRNAs have average expression that is ≥1/1,024-th of ACTB (2^10^ = 1,024). We retained 9,871 transcribed mRNAs. Importantly, we used the mRNA RPKM values that are reported in the RSEM normalized output that is part of the UNC mRNA pipeline of the TCGA data. Then, we examined the correlation between the abundance of these mRNAs and the abundance of isomiRs across all samples. To elucidate potentially meaningful relationships, we limited our focus on the 932 isomiRs that are differentially abundant (according to SAM) between normal and tumor samples at FDR = 0% and have median abundance ≥1 RPM. A first set of 210,682 isomiR-mRNA correlations emerged (Supp. Table 4).

IsomiRs (i.e. miRNAs) interact directly with mRNA transcripts degrading them and leading to a concomitant decrease of protein abundance. Such direct interactions are expected to be captured by negatively-correlated isomiR-mRNA pairs. On the other hand, positive correlations between miRNAs and mRNAs may reflect indirect events downstream of direct targets. In previous work, we showed experimentally that two isomiRs *x* and *y* from the same miRNA arm can differentially impact the abundance of an mRNA *m*: transfection of *x* could lead to a decrease in the abundance of *m*, whereas transfection of *y* could lead to an increase in the abundance of *m*, and vice versa^49^. Thus, we computed both positive and negative correlations and list them in Supp. Table 4. There are 19,812 pairs with a correlation with absolute value ≥ 0.33, including 412 isomiRs and 1,683 mRNAs.

**Figure 7:**
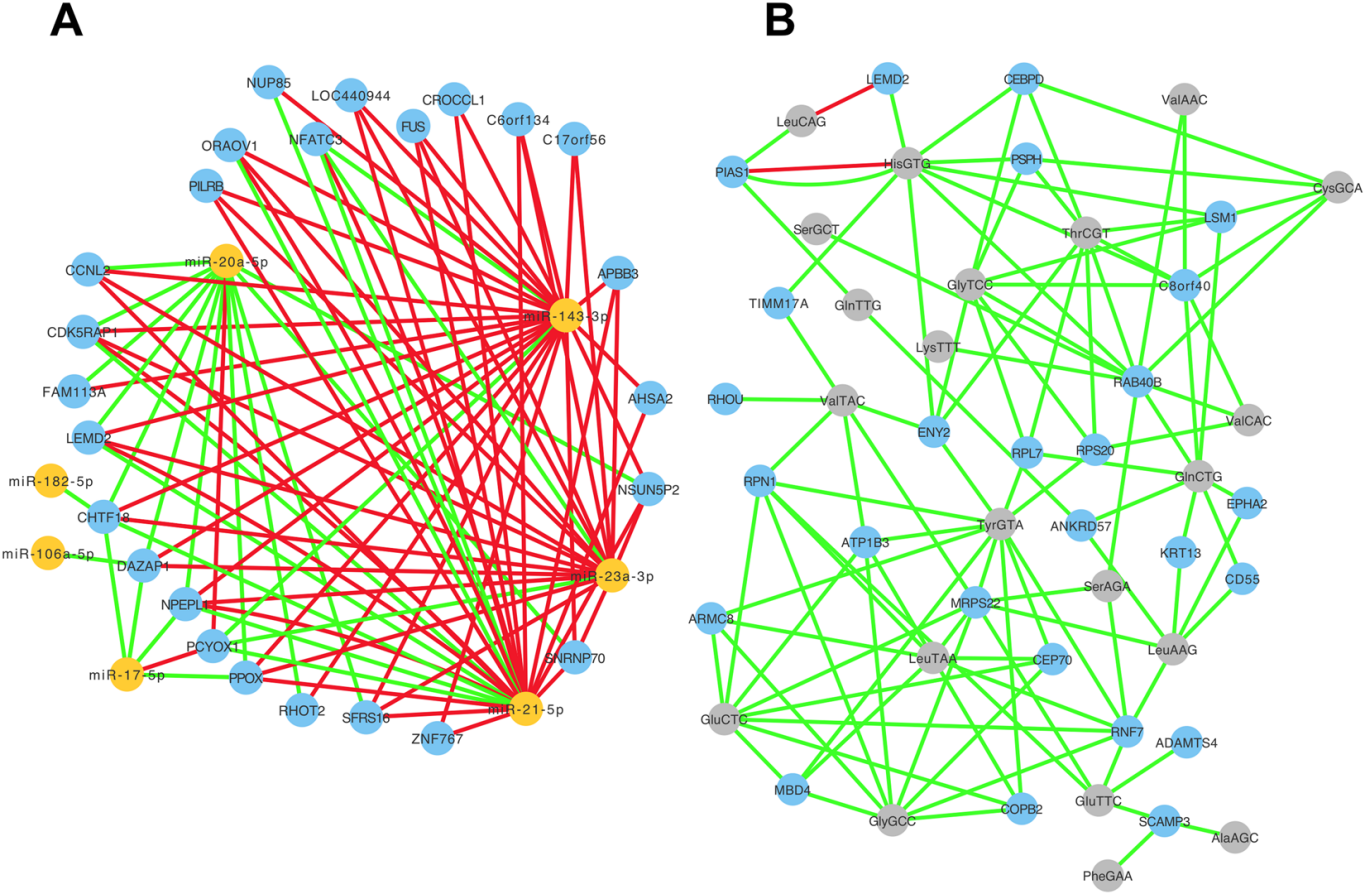
Correlation networks for isomiR-mRNA and tRF-mRNA relationships. We sub-selected differentially abundant isomiRs and tRFs and kept only those with mean abundance ≥1 RPM across all 526 datasets. We computed Spearman correlations isomiRs and tRFs and those mRNAs whose mean RPKM was ≥ 1/1024 of the largest abundance observed for ACTB as measured by sequenced reads. (**A**) isomiR-mRNA correlations. (**B**) tRF-mRNA correlations. In panels A and B, ncRNA nodes were collapsed to the name of the contributing miRNA locus (orange) or tRNA isodecoder (grey), respectively. Blue nodes represent mRNAs. In both panels, only correlations with an absolute value ≥ 0.33 and an FDR ≤ 5% were retained and shown. Green edges: positive correlations. Red edges: negative correlations.

From these correlations with absolute value ≥ 0.33, we sub-selected those from 60 isomiRs from the top five isomiR-producing miRNAs shown in Fig. 1D. We combined these with the 12 isomiRs from the 5 miRNA loci that participate in the denser networks of the miRNA-miRNA interactions shown in Fig. 6A. We then visualize only mRNAs that participate in isomiR-mRNA pairs with ≥15 distinct isomiRs originating from at least two different miRNA loci. Also, we used a single node labeled with the name of the isomiRs’ parental miRNA precursor to capture multiple isomiRs. Figure 7A shows the resulting network, which involves 57 isomiRs from 7 miRNA loci and 27 mRNAs.

For functional enrichment analysis, we concentrated only on negatively-correlated isomiR-mRNA pairs. These pairs were formed by 775 isomiRs and 2,912 mRNAs (Supp. Table 4). Using four different tools (TargetScan^50^, rna22^51^, miRanda^50^, RNAhybrid^52^), we determined which mRNAs were also predicted targets of the isomiRs with which they were anti-correlated. For rna22, miRanda, and RNAhybrid, we generated predictions using the full-length of the isomiRs of interest and the 3′UTRs of the corresponding anti-correlated mRNA. For Targetscan, we used the seed (positions 2–8 of the miRNA) of the corresponding isomiR of interest with the full length of the corresponding anti-correlated mRNA. See Supp. Table 4.

We used the target predictions to sub-select the negatively-correlated isomiR-mRNA pairs. Specifically, we kept only those mRNAs that were also supported by the 4 target-prediction tools. 310 mRNAs survived this filtering (Supp. Table 4). We analyzed them using DAVID and found enrichment for 25 GO terms. The top 5 GO terms include: regulation of nucleobase-containing compound metabolism, DNA binding, regulation of nitrogen compound metabolic process, regulation of macromolecule biosynthetic process, and regulation of cellular macromolecule biosynthetic process.

For comparison, we also investigated how functional enrichments might change if we used the strength of correlation as a filter, instead of target predictions. Doing so retains 754 mRNAs that are negatively correlated with isomiRs at r ≤ −0.33. DAVID analysis revealed 57 GO terms enriched in this group of mRNAs. The corresponding GO terms are listed in Supp. Table 4. The top 5 GO terms include: RNA splicing; mRNA processing; mRNA splicing, via spliceosome; RNA splicing, via transesterification reactions with bulged adenosine as nucleophile; and RNA splicing, via transesterification reactions.

To assess whether race differences could play a part in these dynamics, we focused on the 196 isomiRs that we observed to be differentially abundant between Wh PRAD and B/Aa PRAD (Fig. 4A). We sub-selected among all isomiR-mRNA correlations only those associated with these race-dependent isomiRs at r ≤ −0.33, obtaining 13,230 negatively-correlated isomiR-mRNA pairs. We analyzed the corresponding 93 mRNAs with DAVID and found 42 enriched GO terms (Supp. Table 4). Among these terms, we find enrichment for terms such as: cellular response to hormone stimulus, reproductive process, reproduction, and multicellular organism reproduction.

### Correlation between tRFs and mRNAs

Next, we extended our analysis to correlations involving tRFs and mRNAs. We again correlated the expression of the mRNAs identified above with the expression of tRFs across all samples, using Spearman correlation. We limited our focus to the 192 tRFs that are differentially abundant between normal and tumor samples and have median abundance ≥ 1RPM (Supp. Table 3) and filtered them further retaining only those with absolute value ≥ 0.33 and FDR ≤ 5%. We kept both negative and positive correlations, because the mode in which tRFs function has yet to be understood well. As mentioned in the Introduction, these ncRNAs have been demonstrated to function as miRNAs – suggesting negative correlations would represent functional relationships – but have also been shown to decoy RNA binding proteins – suggesting positive correlations would show functional relationships as well. For presentation purposes, we then collapsed the correlation relationships by replacing individual tRF sequences with the name of the tRNA isoacceptor from which they arise. This left us with 423 correlation pairs (23 isoacceptors and 223 mRNAs) that are listed in Supp. Table 4. In Fig. 7B we depict these pairs. For clarity, we limited our graph and show only those mRNAs that participate in ≥4 correlations. DAVID analysis of the 223 mRNAs that survived the filtering showed 10 enriched GO terms: cellular response to endogenous stimulus, response to endogenous stimulus, response to organic substance, cellular response to hormone stimulus, response to hormone, response to oxygen-containing compound, collagen fibril organization, response to organic cyclic compound, response to organonitrogen compound, and response to nitrogen compound. Thirteen mRNAs were negatively correlated with tRFs (Supp. Table 4). Removing them from the list of 223 mRNAs and repeating the DAVID analysis did not change the list of enriched GO terms. Lastly, we examined the mRNAs that were correlated with the 200 tRFs that are differentially abundant between the Wh PRAD samples and the B/Aa PRAD samples. We did not find any enriched GO terms in this group.

### TCGA PRAD tRFs vs. tRFs obtained from other prostate cancer samples

In previous work^20^, we demonstrated consistent differences in the tRF profiles of individuals that differ by gender, population origin or race. This observation thus becomes an important consideration when comparing tRF profiles from samples of different origin. With that in mind, we sought to compare our TCGA PRAD tRF profiles with those from two different collections of prostate samples for which deep sequencing data is available, but for which neither the population origin nor the race of the patient donors is reported. We note here that, by design, the TCGA repository comprises samples from individuals whose race is self-reported.

The first non-TCGA collection comprises the 11 samples described in earlier work^41^, a study that sought to establish a prostate-cancer-specific tRF signature. An additional characteristic of this collection is that each of its 11 samples was created by pooling together samples from multiple patients. The second non-TCGA collection comprises five normal prostate and five PRAD samples that we generated for a different project^16^ (available at http://trace.ddbj.nig.ac.jp/DRASearch/study?acc = SRP034550). To ensure consistency in the mining steps, we analyzed the 21 samples in these two collections using MINTmap and Threshold-seq (see Materials and Methods), just as we did for the TCGA PRAD datasets.

Our mining of the 11 datasets of the first collection^41^ uncovered 3,341 tRFs above Threshold-seq threshold in at least one of the samples. Of the 6,296 tRFs that we find in the TCGA PRAD collection, 1,856 (29.48%) are also present among the tRFs that MINTmap finds in these 11 samples, and we list them in Supp. Table 5 together with their respective universal identifiers, i.e. their *license plates*, which we described elsewhere^53^. The Table also indicates which of the tRFs are exclusive to tRNA space or can be found in locations of the genome that do not harbor tRNAs.

Our mining of the 10 datasets from our parallel project^16^ uncovered 10,505 tRFs above Threshold-seq’s threshold in at least one of the samples. Of the 6,296 tRFs that we find in the TCGA PRAD collection, 2,090 (33.20%) are also present among the tRFs found in this collection and are listed in Supp. Table 5.

Finally, we compared all three of these collections and found 923 tRFs that are present consistently in all of them. Considering the potential diversity of the patients who contributed all these samples, it is encouraging to see such a high number of tRFs being consistently present across collections. This observation coupled to the demonstrated tissue specificity of tRFs suggests that devising tRF-based biomarkers for this cancer or for other types of cancer is a real possibility.

## Discussion

In recent work, we identified potential roles for miRNAs/isomiRs and tRFs as tissue- and patient-specific post-transcriptional regulators. Here, we characterized the tRF and isomiR profiles of 526 human prostate samples from TCGA and examined whether these molecules exhibit differential abundance in disease compared to normal and whether there is a race-specific component to the abundance of these molecules. Our analyses also investigated the correlations between isomiRs and mRNAs, and between tRFs and mRNAs, to elucidate putative regulatory connections between these short non-coding RNAs and the post-transcriptional regulation layer that is at work in prostate.

Our analyses showed that the TCGA PRAD datasets are characterized by very rich tRF profiles. Interestingly, these profiles are largely similar to tRF profiles that we generated by analyzing two different cohorts^1,41^, suggesting a consistency of tRF profiles in prostate tumors across likely diverse populations. Arguably, the 923 tRFs that we identified as being present in all three cohorts represent a good starting point for researchers interested in studying the roles of these molecules in the prostate context.

DAVID analyses of the mRNAs that are correlated with isomiRs and tRFs reveal interesting potential roles for each of these two classes of regulators in disease. When we filtered our correlations by retaining only those with predicted miRNA target interactions from all 4 different algorithms, our mRNA input list led to an enrichment for GO terms relating to metabolic and biosynthetic processes. When we filtered isomiRs based on the strength of anticorrelation with mRNAs (r ≤ −0.33), pathways that are critical for the production of mature mRNA products were enriched instead, including: mRNA processing, RNA splicing, mRNA splicing via spliceosome, RNA metabolic process, and mRNA metabolic process.

We also find specific GO enrichments for isomiR-mRNA correlations with isomiRs that were differentially expressed between PRAD Wh and PRAD B/Aa samples. Specifically, GO enrichments from these 93 mRNAs pertain to reproductive pathways and are characterized by an overabundance of androgen receptor signaling machinery genes. Future work should look to determine whether isomiRs could play a race-independent role in PRAD biology, as well as whether they could influence the variability of androgen receptor activity seen in different race groups.

For tRFs that are differentially abundant between normal prostate and PRAD (both races), we found that correlated mRNAs enriched in GO terms pertaining to cellular responses to stimuli. These terms include: GO:0071495-cellular response to endogenous stimulus, GO:0009719-response to endogenous stimulus, GO:0010033-response to organic substance, GO:0032870-cellular response to hormone stimulus, and GO:0009725-response to hormone. We note that the large majority of tRFs are positively correlated with mRNAs and that negative correlations between tRFs and mRNAs do not significantly contribute to GO enrichments.

Collectively, these findings raise the possibility of a disease biology in which dysregulation of miRNAs/isomiRs might give rise to accumulating oncogenic transcripts, whereas dysregulation of tRFs might render cells insensitive to control signals in the microenvironment. Specifically, isomiRs may be involved in regulating nucleic acid and protein metabolism or may be involved in regulating splicing machinery. Losing these isomiRs in prostate cancer contexts may contribute to the accumulation of alternatively spliced oncogenic transcripts that both contribute to disease progression and account for some differential characteristics of disease^54,55^. Alternatively, isomiRs may accumulate or be co-regulated with mRNAs involved in upregulation of the proliferative pathways that contribute to tumor biology – i.e., those involved in nuclear division, cell cycle processes, or metabolism (see above). tRFs might be produced in cells actively responding to metabolic changes in the microenvironment or to paracrine/autocrine signaling, whereas isomiRs might become dysregulated in cells experiencing pro-proliferative or cancerous transformations driven by other mechanisms. We note here that tRNA halves have been reported to be produced in response to hormone signaling^23^. Follow-up studies will need to establish causal relationships from these correlations. The investigation of a possible regulatory relationship between these two groups of ncRNAs and prostate cancer specific gene pathways is warranted.

Our findings suggest the possibility of developing a deep-sequencing-based approach that examines the abundance of a specific subset of short ncRNAs and allows the automated detection of prostate cancer. This would enable diagnosis in a manner that is minimally dependent on human observation. It is important to note here that our earlier findings indicate that it may not be feasible to base such a diagnosis on qRT-PCR techniques aimed at estimating the abundance of specific isomiRs or tRFs. Indeed, as we showed, the simultaneous presence of near-identical variants of the targeted isomiR or tRF introduces cross-talk that skews these measurements^48^.

In terms of prioritizing future work, we believe that initial efforts should be directed at elucidating the biogenesis and functional roles of these molecules. Doing so will have a direct beneficial impact when designing experiments to study these molecules. In the absence of this knowledge, it is difficult to know the extent to which attempts to over-express or silence a tRF disrupt other cellular processes leading to unintended consequences.

## Materials and Methods

### Sample Collection and Patient Enrollment

The TCGA initiative enrolled all of the PRAD patients whose samples were analyzed in this presentation. We focused on and analyzed the short RNA profiles from 526 prostate cancer datasets from 472 patients that were part of TCGA PRAD as of October 2015. Only samples that were ‘white-listed’ by the PRAD consortium were analyzed, meaning that these samples did not bear an annotation calling for their exclusion from the TCGA PRAD project. We also included 10 samples from an independent dataset first discussed in^1^, which includes deep sequencing from 5 prostate cancer tumor tissue samples, as well as 5 normal prostate tissue controls. We further included 11 datasets that represent pooled prostate tissue RNA samples from 11 patient groups, including one FFPE tumor preserved sample set and one normal healthy prostate tissue sample set.

### Mapping

For the isomiR analyses, we mapped the reads on the full genome, as we previously described^13,15,20^. For the tRF analyses, we used the MINTmap tool we reported recently^56^ to mine for and quantify tRFs. The mRNA data were generated by the TCGA initiative and downloaded from the *rsem*.*genes*.*normalized_expression* files generated as output of the UNC mRNA analysis pipeline. All mRNA values represent RPKM values as computed by the RSEM RNA-seq analysis platform.

### Data Thresholds and Normalization

After mapping, and for each of the 526 TCGA samples in turn, we thresholded isomiRs and tRFs using the Threshold-seq method^42^. Read counts were normalized to Reads Per Million (RPM) by referring to the number of reads that survive quality trimming and adapter removal.

### Statistical Analyses

We used the R software package for statistical analyses. We used the *prcomp* package for PCA analysis^57^ and the *samr* package for the Significance Analysis of Microarrays^46^. Plots were generated using the *gplots*, *matplotlib* and *igraph* packages.

### miRNA Target Prediction

We used TargetScan 7.1^50^, miRanda^50^, RNAhybrid^52^, and rna22^58^ (http://cm.jefferson.edu/rna22/) to generate miRNA target predictions. We sought targets in the 3′UTRs of mRNAs in all four tools. We used mRNAs and isomiRs that were negatively correlated in our data for predictions in all tools.

### DAVID Functional Analysis

GO term enrichments were computed using DAVID^59^, with an FDR threshold of less than or equal to 1%^59^ (Release 6.8 of October 2016). We performed functional analysis first using mRNAs that are predicted as targets of isomiRs with which they are negatively correlated. We considered only targets predicted by all four prediction tools. For comparison purposes, we also performed functional analysis using those mRNAs that were negatively correlated with isomiRs with correlation value less than or equal to −0.33.

### Data Availability

The TCGA datasets that are described in this study are available from The Cancer Genome Atlas data portal at: https://portal.gdc.cancer.gov/. The deep sequencing datasets from normal prostate and PRAD originally discussed in our previous work^1^ are available at http://trace.ddbj.nig.ac.jp/DRASearch/study?acc = SRP034550. The data of the earlier prostate cancer study^41^ are available through GEO accession GSE80400. All of the data that we generated in our analyses are included in the Supplemental material accompanying this manuscript.

### Declarations

#### Ethics approval and consent to participate

Not applicable for the TCGA datasets. For the other two datasets, see previous publications^1,41^.

### Availability of data and materials

The original datasets that we used and/or analyzed during the current study are available from The Cancer Genome Atlas data portal at: https://portal.gdc.cancer.gov/. The alternative datasets are available through http://trace.ddbj.nig.ac.jp/DRASearch/study?acc = SRP034550 and through GEO accession GSE80400.

## Electronic supplementary material

Supplementary Information

Supplementary Table 1

Supplementary Table 2

Supplemental Table 3

Supplemental Table 4

Supplemental Table 5
