# Supplementary material for: Profiles of miRNA Isoforms and tRNA Fragments in Prostate Cancer
Source: Sci Rep. 2018 Mar 28;8:5314. doi: 10.1038/s41598-018-22488-2 (PMC5871839; doi:10.1038/s41598-018-22488-2)
Supplement: Supplementary file 1 — Supplementary Information [file 41598_2018_22488_MOESM1_ESM.pdf]

Supplementary Information for "Profiles of miRNA Isoforms and tRNA Fragments in Prostate Cancer"

Rogan G. Magee, Aristeidis G. Telonis, Phillipe Loher, Eric Londin, Isidore Rigoutsos*

Computational Medicine Center, Thomas Jefferson University, 1020 Locust Street, Philadelphia, PA 19107.

* Contact: Isidore Rigoutsos, isidore.rigoutsos@jefferson.edu / 215-503 4219

Supplementary Figure 1. Overlap of significant tRFs from three datasets. We gathered significant tRF reads from each of three datasets: 1) TCGA, 2) datasets from Olvedy et al.[1], dubbed “Olvedy”, and 3) datasets from a prior publication from our group[2], dubbed “CMC”. We used the MINTmap tool[3] to gather tRF reads and subjected these reads to Threshold-seq[4], in order to determine which reads surpassed threshold. We show here a Venn diagram depicting the overlap of unique tRF sequences gathered from each of three datasets, for: 1) all samples in each of the datasets, 2) only normal samples in each of the datasets, and 3) only tumor samples in each of the datasets.

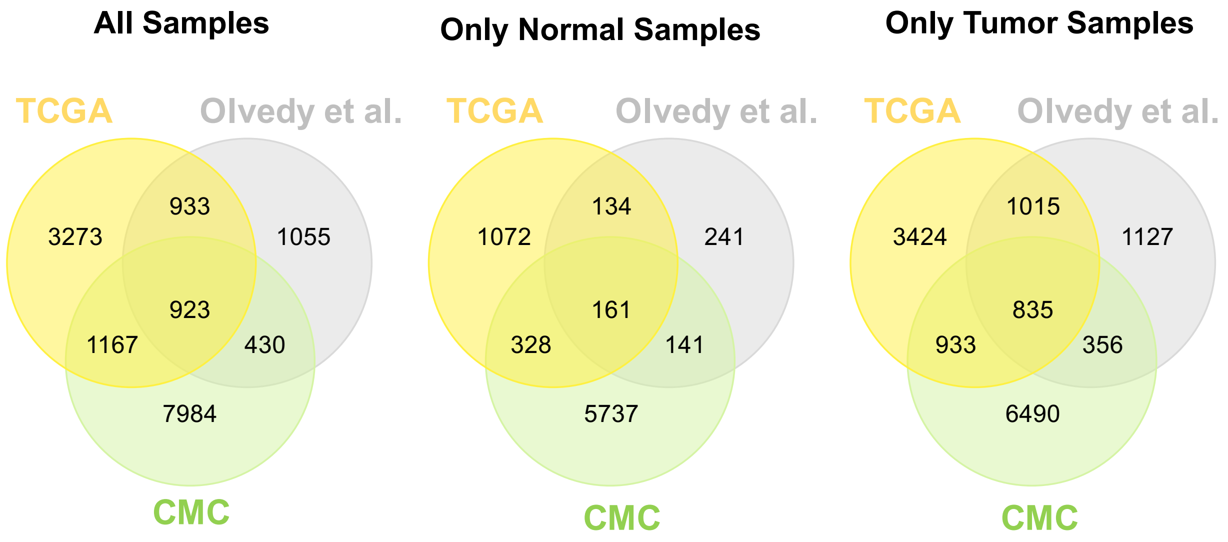

Supplemental Table 1. Mature miRNAs contributing isomiRs. All mature miRNA arms contributing more than one isomiR are annotated in Supp. Table 1. Mature miRNAs are grouped according to the number of mature isomiRs that pass Threshold-seq thresholds in the TCGA PRAD data.

Supplemental Table 2. Differentially abundant isomiRs and tRFs. This Table lists all isomiRs and tRFs that were found to be differentially abundant in the comparisons mentioned throughout the paper. Sheets are separated by FDR (0% or 5%) and by isomiR or tRF.

Supplemental Table 3. IsomiR-isomiR and tRF-tRF correlations. This Table shows Spearman correlation values for miRNA arm-miRNA arm, isomiR-isomiR, tRNA isoacceptor-tRNA isoacceptor, and tRF-tRF pairs, in this order.

Supplemental Table 4. IsomiR-mRNA and tRF-mRNA correlations. This Table shows Spearman correlation values for isomiR-mRNA and tRF-mRNA comparisons. isomiR correlations are listed twice: once at the isomiR level, and a second time at the miRNA-arm level. Similarly, for tRFs and tRNA isoacceptors. Also included in this Table is the output of the DAVID analysis of the mRNAs that are correlated with isomiRs and tRFs, respectively.

Supplemental Table 5. tRFs in prostate samples from other cohorts. This table reports all tRFs found by MINTmap[3] in two additional cohorts: 1) 10 datasets (normal prostate and prostate cancer) originally reported in[2]; and 2) 11 datasets (pooled prostate cancer samples) of[1]. We list the tRF sequence, together with the tRF license plate[5], label within the original publication, and information on whether the tRF is exclusive to tRNA space. We also report the support for each tRF in each sample.

REFERENCES

1 Olvedy, M., *et al*. A Comprehensive Repertoire of tRNA-Derived Fragments in Prostate Cancer. *Oncotarget* **7**, 24766-24777 (2016).

2 Londin, E., *et al.* Analysis of 13 Cell Types Reveals Evidence for the Expression of Numerous Novel Primate- and Tissue-Specific microRNAs. *Proceedings of the National Academy of the Sciences* **112**, E1106-E1115 (2014).

3 Loher, P., Telonis, A. G. & Rigoutsos, I. MINTmap: fast and exhaustive profiling of nuclear and mitochondrial tRNA fragments from short RNA-seq data. *Sci Rep* **7**, 41184, doi:10.1038/srep41184 (2017).

4 Magee, R., Loher, P., Londin, E. & Rigoutsos, I. Threshold-seq: a tool for determining the threshold in short RNA-seq datasets. *Bioinformatics*, doi:10.1093/bioinformatics/btx073 (2017).

5 Pliatsika, V., Loher, P., Telonis, A. G. & Rigoutsos, I. MINTbase: a framework for the interactive exploration of mitochondrial and nuclear tRNA fragments. *Bioinformatics* **32**, 2481-2489, doi:10.1093/bioinformatics/btw194 (2016).
